# Supplementary material for: Predominance of multidrug-resistant Salmonella Typhi genotype 4.3.1 with low-level ciprofloxacin resistance in Zanzibar
Source: PLoS Negl Trop Dis. 2024 Apr 17;18(4):e0012132. doi: 10.1371/journal.pntd.0012132 (PMC11057722; doi:10.1371/journal.pntd.0012132)
Supplement: S1 Table — (DOCX) [file pntd.0012132.s001.docx]

**Supplementary 1 Table. Per-isolate information on AMR genotype profile, year, AMR determinants and accession numbers of genomes (submitted to ENA project PRJEB59168 and to GenBank BioProject PRJNA982791)**

| **ID** | **AMR genotype profile** | **Year** | **Amino-glycoside** | **Beta lactam** | **Phenicol** | **Sulfa-methoxazole, trimethoprim** | **QRDR mutations** | **Accession**  **number**  **short read** | **Accession number**  **long read** |
| --- | --- | --- | --- | --- | --- | --- | --- | --- | --- |
| ZNZ01F8 | MDR/cipR 4.3.1.1 | 2015 | *aph(6)-Id,*  *aph(3'')-Ib* | *bla*_TEM-1B_ | *catA1* | *sul1, sul2, dfrA7* | *gyrA*: p.D87G | ERR11412256 |  |
| ZNZ02F158 | MDR/cipR 4.3.1.1 | 2016 | *aph(6)-Id,*  *aph(3'')-Ib* | *bla*_TEM-1B_ | *catA1* | *sul1, sul2, dfrA7* | *gyrA*: p.D87G | ERR11412266 |  |
| ZNZ03L9 | MDR/cipR 4.3.1.1 | 2015 | *aph(6)-Id,*  *aph(3'')-Ib* | *bla*_TEM-1B_ | *catA1* | *sul1, sul2, dfrA7* | *gyrA*: p.D87G | ERR11270576 |  |
| ZNZ04L15 | MDR/cipS 4.3.1.1 | 2015 | *aph(6)-Id,*  *aph(3'')-Ib* | *bla*_TEM-1B_ | *catA1* | *sul1, sul2, dfrA7* | *-* | ERR11412010 | ERZ18315935 |
| ZNZ05L19 | MDR/cipS 4.3.1.1 | 2015 | *aph(6)-Id,*  *aph(3'')-Ib* | *bla*_TEM-1B_ | *catA1* | *sul1, sul2, dfrA7* | *-* | ERR11412012 |  |
| ZNZ06L25 | MDR/cipR 4.3.1.1 | 2015 | *aph(6)-Id,*  *aph(3'')-Ib* | *bla*_TEM-1B_ | *catA1* | *sul1, sul2, dfrA7* | *gyrA*: p.D87G | ERR11412277 |  |
| ZNZ07L26 | MDR/cipR 4.3.1.1 | 2015 | *aph(6)-Id,*  *aph(3'')-Ib* | *bla*_TEM-1B_ | *catA1* | *sul1, sul2, dfrA7* | *gyrA*: p.D87G | ERR11412278 |  |
| ZNZ08L51 | MDR/cipS 4.3.1.1 | 2015 | *aph(6)-Id,*  *aph(3'')-Ib* | *bla*_TEM-1B_ | *catA1* | *sul1, sul2, dfrA7* | - | ERR11412269 |  |
| ZNZ09L64 | MDR/cipS 4.3.1.1 | 2015 | *aph(6)-Id,*  *aph(3'')-Ib* | *bla*_TEM-1B_ | *catA1* | *sul1, sul2, dfrA7* | - | ERR11412270 |  |
| ZNZ10L65 | MDR/cipR 4.3.1.1 | 2015 | *aph(6)-Id,*  *aph(3'')-Ib* | *bla*_TEM-1B_ | *catA1* | *sul1, sul2, dfrA7* | *gyrA*: p.D87G | ERR11412271 |  |
| ZNZ11L75 | MDR/cipS 4.3.1.1 | 2015 | *aph(6)-Id,*  *aph(3'')-Ib* | *bla*_TEM-1B_ | *catA1* | *sul1, sul2, dfrA7* | - | ERR11414157 |  |
| ZNZ12L76 | MDR/cipS 4.3.1.1 | 2015 | *aph(6)-Id,*  *aph(3'')-Ib* | *bla*_TEM-1B_ | *catA1* | *sul1, sul2, dfrA7* | - | ERR11414158 |  |
| ZNZ13L78 | MDR/cipS 4.3.1.1 | 2015 | *aph(6)-Id,*  *aph(3'')-Ib* | *bla*_TEM-1B_ | *catA1* | *sul1, sul2, dfrA7* | - | ERR11413875 | SAMN35714885 |
| ZNZ14F11 | MDR/cipR 4.3.1.1 | 2015 | *aph(6)-Id,*  *aph(3'')-Ib* | *bla*_TEM-1B_ | *catA1* | *sul1, sul2, dfrA7* | *gyrA*: p.D87G | ERR11414161 |  |
| ZNZ15F16 | MDR/cipR 4.3.1.1 | 2015 | *aph(6)-Id,*  *aph(3'')-Ib* | *bla*_TEM-1B_ | *catA1* | *sul1, sul2, dfrA7* | *gyrA*: p.D87G | ERR11413762 |  |
| ZNZ16F33m | MDR/cipS 4.3.1.1 | 2015 | *aph(6)-Id,*  *aph(3'')-Ib* | *bla*_TEM-1B_ | *catA1* | *sul1, sul2, dfrA7* | - | ERR11414162 |  |
| ZNZ17F60 | MDR/cipR 4.3.1.1 | 2015 | *aph(6)-Id,*  *aph(3'')-Ib* | *bla*_TEM-1B_ | *catA1* | *sul1, sul2, dfrA7* | *gyrA*: p.D87G | ERR11414163 | SAMN35713968 |
| ZNZ18F80 | MDR/cipR 4.3.1.1 | 2015 | *aph(6)-Id,*  *aph(3'')-Ib* | *bla*_TEM-1B_ | *catA1* | *sul1, sul2, dfrA7* | *gyrA*: p.D87G | ERR11414164 |  |
| ZNZ19F87 | MDR/cipR 4.3.1.1 | 2016 | *aph(6)-Id,*  *aph(3'')-Ib* | *bla*_TEM-1B_ | *catA1* | *sul1, sul2, dfrA7* | *gyrA*: p.D87G | ERR11412666 |  |
| ZNZ20F88 | MDR/cipR 4.3.1.1 | 2016 | *aph(6)-Id,*  *aph(3'')-Ib* | *bla*_TEM-1B_ | *catA1* | *sul1, sul2, dfrA7* | *gyrA*: p.D87G | ERR11414165 |  |
| ZNZ21F90 | MDR/cipR 4.3.1.1 | 2016 | *aph(6)-Id,*  *aph(3'')-Ib* | *bla*_TEM-1B_ | *catA1* | *sul1, sul2, dfrA7* | gyrA: p.D87G | ERR11412281 |  |
| ZNZ22F108 | MDR/cipR 4.3.1.1 | 2016 | *aph(6)-Id,*  *aph(3'')-Ib* | *bla*_TEM-1B_ | *catA1* | *sul1, sul2, dfrA7* | gyrA: p.D87G | ERR11414242 |  |
| ZNZ23F110 | MDR/cipR 4.3.1.1 | 2016 | *aph(6)-Id,*  *aph(3'')-Ib* | *bla*_TEM-1B_ | *catA1* | *sul1, sul2, dfrA7* | *gyrA*: p.D87G | ERR11413529 |  |
| ZNZ24F129 | MDR/cipR 4.3.1.1 | 2016 | *aph(6)-Id,*  *aph(3'')-Ib* | *bla*_TEM-1B_ | *catA1* | *sul1, sul2, dfrA7* | *gyrA*: p.D87G | ERR11414243 |  |
| ZNZ25L95 | MDR/cipS 4.3.1.1 | 2015 | *aph(6)-Id,*  *aph(3'')-Ib* | *bla*_TEM-1B_ | *catA1* | *sul1, sul2, dfrA7* | - | ERR11414244 |  |
| ZNZ26L100 | MDR/cipS 4.3.1.1 | 2015 | *aph(6)-Id,*  *aph(3'')-Ib* | *bla*_TEM-1B_ | *catA1* | *sul1, sul2, dfrA7* | *-* | ERR11412282 |  |
| ZNZ27L145 | MDR/cipR 4.3.1.1 | 2016 | *aph(6)-Id,*  *aph(3'')-Ib* | *bla*_TEM-1B_ | *catA1* | *sul1, sul2, dfrA7* | *gyrA*: p.D87G | ERR11414246 |  |
| ZNZ28L107 | MDR/cipS 4.3.1.1 | 2015 | *aph(6)-Id,*  *aph(3'')-Ib* | *bla*_TEM-1B_ | *catA1* | *sul1, sul2, dfrA7* | - | ERR11414247 |  |
| ZNZ29L147 | MDR/cipR 4.3.1.1 | 2016 | *aph(6)-Id,*  *aph(3'')-Ib* | *bla*_TEM-1B_ | *catA1* | *sul1, sul2, dfrA7* | *gyrA*: p.D87G | ERR11412670 |  |
| ZNZ30L150 | MDR/cipR 4.3.1.1 | 2016 | *aph(6)-Id,*  *aph(3'')-Ib* | *bla*_TEM-1B_ | *catA1* | *sul1, sul2, dfrA7* | *gyrA*: p.D87G | ERR11414248 |  |
| ZNZ31L191 | MDR/cipR 4.3.1.1 | 2016 | *aph(6)-Id,*  *aph(3'')-Ib* | *bla*_TEM-1B_ | *catA1* | *sul1, sul2, dfrA7* | *gyrA* p.D87G | ERR11414250 |  |
| ZNZ32L209 | MDR/cipR 4.3.1.1 | 2016 | *aph(6)-Id,*  *aph(3'')-Ib* | *bla*_TEM-1B_ | *catA1* | *sul1, sul2, dfrA7* | *gyrA*: p.D87G | ERR11414251 |  |
| ZNZ33L223 | MDR/cipS 4.3.1.1 | 2016 | *aph(6)-Id,*  *aph(3'')-Ib* | *bla*_TEM-1B_ | *catA1* | *sul1, sul2, dfrA7* | - | ERR11414252 |  |
| ZNZ34L232 | MDR/cipS 4.3.1.1 | 2016 | *aph(6)-Id,*  *aph(3'')-Ib* | *bla*_TEM-1B_ | *catA1* | *sul1, sul2, dfrA7* | - | ERR11414253 |  |
| ZNZ35L233 | MDR/cipS 4.3.1.1 | 2016 | *aph(6)-Id,*  *aph(3'')-Ib* | *bla*_TEM-1B_ | *catA1* | *sul1, sul2, dfrA7* | - | ERR11413528 |  |
| ZNZ36L257 | MDR/cipR 4.3.1.1 | 2016 | *aph(6)-Id,*  *aph(3'')-Ib* | *bla*_TEM-1B_ | *catA1* | *sul1, sul2, dfrA7* | *gyrA*: p.D87G | ERR11414254 |  |
| ZNZ37M7 | MDR/cipR 4.3.1.1 | 2015 | *aph(6)-Id,*  *ph(3'')-Ib* | *bla*_TEM-1B_ | *catA1* | *sul1, sul2, dfrA7* | *gyrA*: p.D87G | ERR11414255 |  |
| ZNZ38M8 | MDR/cipR 4.3.1.1 | 2015 | *aph(6)-Id,*  *aph(3'')-Ib* | *bla*_TEM-1B_ | *catA1* | *sul1, sul2, dfrA7* | *gyrA*: D87G | ERR11414257 |  |
| ZNZ39M11 | MDR/cipR 4.3.1.1 | 2015 | *aph(6)-Id,*  *aph(3'')-Ib* | *bla*_TEM-1B_ | *catA1* | *sul1, sul2, dfrA7* | *gyrA*: p.D87G | ERR11414258 |  |
| ZNZ40M13 | MDR/cipR 4.3.1.1 | 2015 | *aph(6)-Id,*  *aph(3'')-Ib* | *bla*_TEM-1B_ | *catA1* | *sul1, sul2, dfrA7* | *gyrA*: p.D87G | ERR11412669 |  |
| ZNZ41M30 | MDR/cipR 4.3.1.1 | 2015 | *aph(6)-Id,*  *aph(3'')-Ib* | *bla*_TEM-1B_ | *catA1* | *sul1, sul2, dfrA7* | *gyrA*: p.D87G | ERR11414353 |  |
| ZNZ42M33 | MDR/cipR 4.3.1.1 | 2015 | *aph(6)-Id,*  *aph(3'')-Ib* | *bla*_TEM-1B_ | *catA1* | *sul1, sul2, dfrA7* | *gyrA*: p.D87G | ERR11414354 |  |
| ZNZ43M44 | MDR/cipR 4.3.1.1 | 2015 | *aph(6)-Id*  *aph(3'')-Ib* | *bla*_TEM-1B_ | *catA1* | *sul1, sul2, dfrA7* | *gyrA*: p.D87G | ERR11412654 |  |
| ZNZ44M75 | MDR/cipR 4.3.1.1 | 2015 | *aph(6)-Id,*  *aph(3'')-Ib* | *bla*_TEM-1B_ | *catA1* | *sul1, sul2, dfrA7* | *gyrA*: p.D87G | ERR11413527 |  |
| ZNZ45M81 | MDR/cipS 4.3.1.1 | 2015 | *aph(6)-Id,*  *aph(3'')-Ib* | *bla*_TEM-1B_ | *catA1* | *sul1, sul2, dfrA7* | - | ERR11414355 |  |
| ZNZ46M103 | MDR/cipR 4.3.1.1 | 2015 | *aph(6)-Id,*  *aph(3'')-Ib* | *bla*_TEM-1B_ | *catA1* | *sul1, sul2, dfrA7* | *gyrA*: p.D87G | ERR11414356 |  |
| ZNZ47M104 | MDR/cipR 4.3.1.1 | 2015 | *aph(6)-Id,*  *aph(3'')-Ib* | *bla*_TEM-1B_ | *catA1* | *sul1, sul2, dfrA7* | *gyrA*: p.D87G | ERR11414358 |  |
| ZNZ48M109 | MDR/cipR 4.3.1.1 | 2015 | *aph(6)-Id,*  *aph(3'')-Ib* | *bla*_TEM-1B_ | *catA1* | *sul1, sul2, dfrA7* | *gyrA*: p.D87G | ERR11414359 |  |
| ZNZ49M111 | MDR/cipR 4.3.1.1 | 2015 | *aph(6)-Id,*  *aph(3'')-Ib* | *bla*_TEM-1B_ | *catA1* | *sul1, sul2, dfrA7* | *gyrA*: p.D87G | ERR11412667 |  |
| ZNZ50M123 | Non MDR/cipR 4.3.1.2 | 2016 |  |  |  |  | ***gyrA*: p.S83F** | ERR11414360 | ERZ18316203 |
| ZNZ51M127 | MDR/cipR 4.3.1.1 | 2016 | *aph(6)-Id,*  *aph(3'')-Ib* | *bla*_TEM-1B_ | *catA1* | *sul1, sul2, dfrA7* | *gyrA* : p.D87G | ERR11414919 |  |
| ZNZ52M130 | MDR/cipR 4.3.1.1 | 2016 | *aph(6)-Id,*  *aph(3'')-Ib* | *bla*_TEM-1B_ | *catA1* | *sul1, sul2, dfrA7* | *gyrA*: p.D87G | ERR11415478 |  |
| ZNZ53M131 | MDR/cipR 4.3.1.1 | 2016 | *aph(6)-Id,*  *aph(3'')-Ib* | *bla*_TEM-1B_ | *catA1* | *sul1, sul2, dfrA7* | *gyrA*: p.D87G | ERR11416037 |  |
| ZNZ54M132 | MDR/cipR 4.3.1.1 | 2016 | *aph(6)-Id,*  *aph(3'')-Ib* | *bla*_TEM-1B_ | *catA1* | *sul1, sul2, dfrA7* | *gyrA*: p.D87G | ERR11416596 |  |
| ZNZ55M142 | MDR/cipS 4.3.1.1 | 2016 | *aph(6)-Id*  *aph(3'')-Ib* | *bla*_TEM-1B_ | *catA1* | *sul1, sul2, dfrA7* | - | ERR11413524 | SAMN35714917 |
| ZNZ56M146 | MDR/cipR 4.3.1.1 | 2016 | *aph(6)-Id,*  *aph(3'')-Ib* | *bla*_TEM-1B_ | *catA1* | *sul1, sul2, dfrA7* | *gyrA*: p.D87G | ERR11413526 |  |
| ZNZ57M188 | MDR/cipR 4.3.1.1 | 2016 | *aph(6)-Id,*  *ph(3'')-Ib* | *bla*_TEM-1B_ | *catA1* | *sul1, sul2, dfrA7* | *gyrA*: p.D87G | ERR11411994 | SAMN35714939 |
| ZNZ58M225 | MDR/cipR 4.3.1.1 | 2016 | *aph(6)-Id,*  *aph(3'')-Ib* | *bla*_TEM-1B_ | *catA1* | *sul1, sul2, dfrA7* | *gyrA*: p.D87G | ERR11416599 |  |
| ZNZPi017 | MDR/cipS 4.3.1.1 | 2012 | *aph(6)-Id,*  *aph(3'')-Ib* | *bla*_TEM-1B_ | *catA1* | *sul1, sul2, dfrA7* | - | ERR11411976 | ERZ18315661 |
| ZNZPi228 | MDR/cipS 4.3.1.1 | 2012 | *aph(6)-Id,*  *aph(3'')-Ib* | *bla*_TEM-1B_ | *catA1* | *sul1, sul2, dfrA7* | - | ERR11412001 | ERZ18315814 |
| ZNZPi450 | MDR/cipS 4.3.1.1 | 2013 | *aph(6)-Id,*  *aph(3'')-Ib* | *bla*_TEM-1B_ | *catA1* | *sul1, sul2, dfrA7* | - | ERR11411981 | ERZ18315820 |

MDR/cipR sub-lineage of 4.3.1.1 = MDR/cipR 4.3.1.1;

MDR/cipS sub-lineage of 4.3.1.1=MDR/cipS 4.3.1.1
