# Supplementary material for: Predominance of multidrug-resistant Salmonella Typhi genotype 4.3.1 with low-level ciprofloxacin resistance in Zanzibar
Source: PLoS Negl Trop Dis. 2024 Apr 17;18(4):e0012132. doi: 10.1371/journal.pntd.0012132 (PMC11057722; doi:10.1371/journal.pntd.0012132)
Supplement: Attachment — Submitted filename: Response_to_reviewers_Typhoid_in_Zanzibar.pdf [file pntd.0012132.s002.pdf]

Responses to Reviewer's Questions and Comments

Comment by the authors: the pages and lines refer to the revised no track changes version of the manuscript.

Methods

Comments by Reviewer #1:

a) This article focuses on the genetic makeup of MDR S.typhi. the topic is relevant and of much importance at global level. The objectives are clear and well-articulated. the study design is fine but lacks details about the pilot project from where historical samples were assessed and compared.

Response: Our response: We thank Reviewer #1 for this important comment
We have added information of the pilot study on page 8 line 141 as follows:
"In addition, the three accessible (of in total seven) *S.* Typhi strains isolated during the pilot study in the years 2012 to 2013 (1) were included in the analyses. The details of the methodology of pilot study have been previously described (1). Briefly, it was performed at the same departments of Mnazi Mmoja Hospital as the main study, whereas 469 participants (neonates, children and adults) presenting with signs of systemic infections were included. As for the present study, the pilot study included clinical data, and blood was collected for culture and susceptibility testing of isolated microbes (1)." We have also added the reference 31 (Onken 2015 (1)) in line 142 and 144.

Comments by Reviewer #1:

b) The isolates included in this analysis are from 2016, would have been nice to analyze the currently prevailing isolates, its almost 8 years old data. Nevertheless, the information adds value to the scientific literature. Appropriate Ethical compliance is in place.

Response: We thank reviewer #1 for this important comment. We fully agree that it is a weakness of the study that the data are eight years old. It would be highly interesting to analyze currently prevailing isolates. However, as there are no newer

isolates accessible, it would require a new study and a considerable amount of time to collect and to analyze newer isolates. Furthermore, to our knowledge, the hospital microbiology laboratory is currently not performing bacterial culture and susceptibility testing.
Yet, our data on isolates from 2016 will add important knowledge both locally and globally. We think that it is important to publish the existing data now both to inform the local and global medical community and decision makers, and to highlight the need of further research. The genomes of the study isolates are uploaded to public accessible databases and can be used for further studies

Comments by Reviewer #2: *The objectives are clear, and the study design and methodology are appropriate and well described.*

Response: Thank you for the positive comment.

<u>Results</u>

Comments by Reviewer #1: *the analysis is quite complete and well presented. Tables and Fig are well presented with detailed legends appropriate for the scientific audience.*

Response: We thank the reviewer for the comment.

Comments by Reviewer #2:

1. a) The results match the aims and methods, however I found the description of the key findings on population structure very hard to follow. In particular the discussion of the various groups very hard to follow, because there is no one table/figure/paragraph that links the 'group' labels; genotypes; and AMR.

Response: Thank you for this comment, to clarify our results we have revised Table 1 to include year of isolation and *S*. Typhi genotypes.

Comments by Reviewer #2:

1. b) Also because the tree topologies are not rooted correctly, it is hard to see but I think the cluster described as 'group A' is actually a monophyletic lineage emerging

from within ‘group B’.

Reading closely and looking at the figures, I think the population structure could be defined much more simply and clearly as follows:

- One isolate (ZNZ50M123) is genotype 4.3.1.2, this has reduced susceptibility to ciprofloxacin (due to gyrA-S83F), and no acquired resistance genes.

- All other isolates were genotype 4.3.1.1, these were all MDR (due to blaTEM-1, catA1, sul1, sul2, dfrA7)

- The majority of these belonged to a monophyletic clade (purple in tree) that had reduced susceptibility to ciprofloxacin (due to gyrA-D87G).

- The others (yellow in tree) include the older isolates from the pilot study, are fully sensitive to ciprofloxacin, and lack QRDR mutations.

Adding the genotypes and years to Table 1 would further clarify; as would rooting the trees properly to highlight the nature of the MDR/CipR sublineage better.

In my view, this provides greater clarity for readers in fewer words, and without needing to introduce arbitrary subgroup labels (group C is a single isolate, so does not need a name; group A can be described as “the local MDR/cipR sub lineage of 4.3.1.1”, which you could give a label to if you really want to; group B is just the rest, it is not monophyletic and doesn’t really need a name (if wanting to refer to these if could be described simply as “MDR/cipS 4.3.1.1”.

I would strongly encourage the authors to rework the manuscript to consider these points.

Response: Thank you for these insightful comments. We fully agree with the assessment. As suggested by the reviewer, we revised the text of the phylogenetic results pages 18-19 line 339 to line 359 and figure legends as follows:

“Figure 2A is a whole genome SNP-based phylogenetic tree containing the 61 *S.* Typhi isolates from this study and one reference strain ERL12960. As shown in Figure 2A, one isolate ZNZ50M123 (ENA accession numbers ERR11414360 and ERZ18316203, project PRJEB59168), highlighted in green belongs to genotype 4.3.1.2. This isolate has reduced susceptibility to ciprofloxacin (due to *gyrA*-S83F), and no acquired resistance genes. All other isolates were genotype 4.3.1.1, these were all MDR (due to bla_{TEM-1}, *catA1*, *sul1*, *sul2*, *dfrA7*). The majority of these belonged to a monophyletic clade (highlighted purple in tree) and had reduced susceptibility to ciprofloxacin (due to *gyrA*-D87G), herein referred as MDR/cipR. In

contrast, the others (highlighted yellow in tree) including the older isolates from the pilot study, are fully sensitive to ciprofloxacin, and lack QRDR mutations referred as MDR/cipS. Within the study isolates, the SNP difference ranged between 0 and 41 (median 7.5), with the biggest SNP between the only isolate belonging to the 4.3.1.2 genotype (ZNZ50M123) and an isolate which is MDR/cipR. For the genomes belonging to the 4.3.1.1 genotype, the overall difference of all study isolates ranged between 0 and 27 SNPs (median 7), within MDR/cipR isolates between 0 and 16 SNPs (median 6), within MDR/cipS isolates between 3 and 17 SNPs (median 9.5). The MDR/cipS isolates had a minimum difference of 11 SNPs to the closest neighbor of MDR/cipR isolates, with two of the pilot study isolates being closest related to MDR/cipR isolates. The only isolate of the genotype 4.3.1.2 (ZNZ50M123), had a difference between 25 and 41 SNPs (median 34) to the other study isolates (all belonging to 4.3.1.1 genotype)."

Capture Figure 2A line 360-373: "**Fig 2A. Phylogenetic tree of *S.* Typhi isolates from this study using the reference genome ERL 12960 as midpoint root.** The inner circle shows three clusters of our *S*. Typhi: purple highlighted is a monophyletic clade (MDR/cipR), these are MDR and have reduced susceptibility to ciprofloxacin due to gyrA-D87G; yellow highlighted are strains which are MDR but sensitive to ciprofloxacin (MDR/cipS); one strain in green highlight is not MDR and has low-level ciprofloxacin resistance due to single *gyrA*-S83F QRDR mutation. The second circle shows the year of isolation of *S*. Typhi; black represents *S.* Typhi isolated in 2016, grey in 2015, light grey and very light grey were isolated in 2013 and 2012 respectively. The next circle shows the season when *S.* Typhi were isolated, green represents isolates collected during the rainy season while orange highlighted *S*. Typhi were collected during the dry season. The outer circle shows the outcome of the patients; red represents *S.* Typhi from patients who died, blue represents *S.* Typhi from patients who survived while grey represents *S.* Typhi from patients with unknown outcome."

Line 378: "to MDR/cipR group (genotype 4.3.1.1, MDR, and *gyrA*-D87G QRDR mutation)"

Capture Figure 2B, line 386-392: "**Fig 2B. Phylogenetic tree of *S.* Typhi isolates from this study and of isolates from other publications using the reference**

genome ERL 12960 as midpoint root. From inside out, the inner circle shows the year of isolation of *S*. Typhi. The second circle shows the country where the strains were isolated. The next circle shows the genotype of the *S.* Typhi strains. The outer circle shows the AMR profile in the current study. The monophyletic MDR/cipR is highlighted in purple, the MDR/cipS cluster is yellow, and the only nonMDR/cipR strain is highlighted in green."

Line 394: "Isolates which are MDR/cipS had"

Line 399: "*gyrA*-S83F ZNZ50M123 had a"

Line 449: "…belonging to a monophyletic clade (MDR/cipR)…"

Line 453: "belonging to the 4.3.1.2 genotype"

Line 483-496: "In the phylogenetic comparison of the study isolates, monophyletic clade (MDR/cipR) isolates showed no SNP difference to the genotypes of *S.* Typhi isolated from two travelers returning to the United Kingdom from Tanzania during the same period (2) . They additionally share the same determinants coding for AMR including the identical *gyrA* point mutation, which, to our knowledge, has not been described in other *S.* Typhi isolates from East Africa. Isolates which are MDR/cipS, are closely related to other strains from Tanzania. Compared to MDR/cipR isolates, MDR/cipS has a smaller SNP difference to isolates from both Kenya and, especially, to those from Zambia. Assessing the results of the epidemiological and the antimicrobial resistance analyses, we speculate that the monophyletic clade (MDR/cipR) isolates may represent a new subtype and an outbreak strain, whereas MDR/cipS may be endemic.

The only non-MDR study strain is belonging to the genotype 4.3.1.2 and harboring a *gyrA*-S83F point mutation. It is closest related to a Tanzanian isolate from 2009 which had the same point mutation but differed"

We have replaced figure 2B by a new version using the reference genome ERL 12960 as a midpoint root. We have revised the color range of the years as suggested by Reviewer #2, however, because of the large number of years, we used three color scales for the year of isolation as seen in the revised Figure 2B. The outer circle is now showing the AMR profiles in the current study, applying the same color scheme as in Figure 2A.

Comment Reviewer #2

2. Table 2 lists all AMR determinants in all 65 genomes (ie one row per isolate), this large table is useful but could be moved to Supplementary and replaced with a summary table in the main text, with one row per AMR genotype profile. The per-isolate table would benefit from adding genotype, accession, year, etc

Response: as suggested by the reviewer, we have revised Table 2. It now shows the distribution of AMR determinants including QRDR mutations as well as *S*. Typhi genotypes with one row per AMR genotype profile. We have moved the per-isolate table to the Supplementary and combined it with the existing Supplementary Table 1 on accession numbers. This revised Supplementary Table 1 shows now genotype, AMR genotype profile, AMR determinant, year and accession number to GenBank and ENA.

Comment Reviewer #2

3. The results concerning integration of MDR are well described except that the authors don't say what the integration site is - ie where in the chromosome is it integrated. Is it one of the previously described sites, or a novel one?

Response: Thank you for the comment, we detected chromosomal integration of the IS*1* at the known site downstream of *cyaY* as previously described (3). We have added this information in the results section on page number 17, line number 331-332. We have also added the reference 22, Hendriksen 2015 (3))

Comment Reviewer #2

4. Lines 334-336, "No plasmid was found, but the MDR S. Typhi strains contained the IncQ1 plasmid replicon sequence (repA and repC) as shown in Figure 1. These are probable remnants of IncHI1 integrated in the chromosome." - the repA/repC is a part of the composite transposon it is not derived from IncHI1

Response: we agree with the reviewer, our MDR S.Typhi strains contained IncQ1 plasmid replicon sequence (repA and repC) which is part of the composite transposon. We have therefore deleted the statement "these are probable remnants of IncHI1 integrated in the chromosome" (line 330).

Comment Reviewer #2

5. How are Figures 2A and 2B rooted? The topologies are very hard to see with the current rooting and plotting choices. Standard practice is to use an outgroup root, or midpoint root; either would solve the issue in this case.
Response: Thank you for your observation, we have revised Figure 2A and 2B. They have been rooted at the midpoint using the reference genome ERL 12960. In the figures, the reference strain is highlighted by using bold text.

Comment Reviewer #2
6. Figure 2B - the colour palettes here are very hard to follow. Suggest changing the years to a greyscale (lighter = earlier, darker = later) as was done in Figure 2A.

Response: We have revised the colour range as suggested; however, because of the large number of years of isolation, we used a three colour scale for the year of isolation as seen in the revised Figure 2B.

Conclusions
Comments by Reviewer #1*: yes, the conclusions are supported by the data presented in the result section*.

Comments by Reviewer #2*: The conclusions are well summarised and supported by the data.*

Editorial and Data Presentation Modifications?

Reviewer #1*: some details regarding the pilot project must be mentioned for readers to know the relevance. need to mention why recent isolates were not included in the analysis. data comprises of isolates from 2015-2016, that is almost 8 years old data. data linked to recent isolates would make it more valuable.*

Response: We have added information of the pilot study on page 8 line 141 as follows:

“In addition, the three accessible (of in total seven) *S.* Typhi strains isolated during the pilot study in the years 2012 to 2013 (1) were included in the analyses. The details of the methodology of pilot study have been previously described (1). Briefly, the pilot study was performed at the same departments of Mnazi Mmoja Hospital as the main study, whereas 469 participants (neonates, children and adults) presenting with signs of systemic infections were included. As for the present study, the pilot study included clinical data and blood was collected for culture and susceptibility testing of isolated microbes (1).”
We have also added the reference 31 (Onken 2015 (1)) in line 142 and 144.
Our data on isolates from 2016 will add important knowledge both locally and globally. We think that it is important to publish the existing data now both to inform the local and global medical community and decision makers and to highlight the need of further research.

Reviewer #2: *(No Response)*

Summary and General Comments

Comments by Reviewer #1: *good work relevant to the local community and globally as the MDR and XDR S.typhi continue to rise, molecular epidemiology is important in tapping the movements of these bugs and assessing the regional genetic mutation pressure. weakness: data is 8 years old, addition of current isolates to the analysis will add value to the data and publication.*
Response: thank you for this important observation. Ideally, we would have liked to include newer data, but we do not have the opportunity to do it at the moment. To our knowledge, the hospital microbiology laboratory is currently not performing bacterial culture and susceptibility testing. Still, we believe that our data on isolates from 2016 will add important knowledge both locally and globally. We think that it is important to publish the existing data now both to inform the local and global medical community and decision makers and to highlight the need of further research.

Comments by Reviewer #2: *The article is overall well written and presents novel data on the Typhi pathogen population responsible for typhoid fever in Zanzibar. The*

methods for characterisation (AST, WGS) are state of the art and fully described. Importantly, the authors place their findings regarding the Zanzibar population in the global context, by comparison with public genomic data.

I noted two issues that should be addressed in the Introduction:

1. Lines 99-101 refer to prior work describing the global population structure of Typhi, however the papers referenced are nearly a decade old (2015/16), suggest citing the 2023 update from the Global Typhoid Genomics Consortium which describes 13,000 genomes (Carey et al 2023, eLife https://doi.org/10.7554/eLife.85867)

Response: Thank you for suggesting this important reference. we have added the reference as suggested, it is now reference number 17, added in line 90, 92, 95.

Comments by Reviewer #2:

2. Line 105, "MDR in S. Typhi is linked to the presence of an integrative conjugative element (ICE)." This statement is not supported by a reference and is incorrect, to my knowledge. MDR in Typhi is associated with a transposon, mobilised by IS1 via a non-conjugative mechanism. The transposon often resides on a conjugative plasmid, but is not itself a conjugative element.

Response: we have revised the text as suggested, on page 6 line 96-99. Now it reads

"MDR in *S*. Typhi is linked to the presence of a composite transposon. In the 4.3.1. genotype, it was first introduced via the IncHI1-PST6 plasmid (4-7). Later, reports have shown that the transposon carrying the genes associated with MDR was integrated into the *S*. Typhi chromosome (3, 7, 8)."

We have also added several references in this section (listed below in "References").

References

Please review your reference list to ensure that it is complete and correct. If you have cited papers that have been retracted, please include the rationale for doing so in the manuscript text, or remove these references and replace them with relevant current references. Any changes to the reference list should be mentioned in the rebuttal letter that accompanies your revised manuscript. If you need to cite a

retracted article, indicate the article's retracted status in the References list and also include a citation and full reference for the retraction notice.

Added references:

Carey 2023, reference 17 line 90, 92,95 (9)
Location of genes coding for AMR:
Holt 2011, reference 21 line 97, 102 (6)
Wong 2015, reference 18, in the previous version already mentioned, now added in the lines 97, 99, 102 (7)
Pilot study: Onken 2015, reference 31, in the previous version already mentioned in line 148, now added in line 142 and line 144 (1)
Chromosomal insertion site of the IS*1*: Hendriksen 2015, reference 22, in the previous version already mentioned, now added in line 332 (3)
Treatment failure when using ciprofloxacin in low-grade ciprofloxacin resistant *S.* Typhi: Threlfall 1999, reference 53, line 434 (10).

Other changes of the manuscript:
Abstract, line 35: rephrased "Typhoid fever is a common cause of febrile illness"
Author summary, lines 59-65: rephrased "spreading globally. Local and regional surveillance of MDR *S*. Typhi populations using both blood culture and whole genome sequencing can uncover outbreaks and help mapping the spread of *S.* Typhi and resistance mechanisms, which, in turn can guide both control and prevention efforts and clinical management. Data regarding the distribution of MDR *S.* Typhi genotypes and resistance mechanisms is scarce in Zanzibar, Tanzania, as in many other African countries. In this study we characterize *S.* Typhi phenotypically and genotypically. This study shows a high rate of MDR *S.* Typhi"
Introduction, line 117 rephrased: "cultures for diagnostic confirmation"
Line 459-460 rephrased: "introducing and spreading azithromycin-resistant *S*. Typhi in the African…"
We have added information on funding line 544-559: " The study received funding from the Norwegian National Centre on Tropical Infectious Diseases, Haukeland University Hospital, Bergen, Norway. Additional support was given by Vestre Viken Hospital Trust, Drammen, Norway, by CAMRIA - Combatting Anti-Microbial

Resistance with Interdisciplinary Approaches, Centre for Antimicrobial Resistance in Western Norway, funded by Trond Mohn Foundation, grant number TMS2020TMT11, and by STRESST - Antimicrobial Stewardship in Hospitals, Resistance Selection and Transfer in a One Health Context, University of Bergen funded by JPIAMR grant number NFR333432.
AO has been granted a PhD scholarship by the Western Norway Regional Health Authority, University of Bergen (Project Number 912277) and has received a salary from Vestre Viken Hospital Trust, Drammen, Norway. SM has received a salary from STRESST - Antimicrobial Stewardship in Hospitals, Resistance Selection and Transfer in a One Health Context, University of Bergen funded by JPIAMR grant number NFR333432.
The funders had no role in study design, data collection and analysis, decision to publish, or preparation of the manuscript."
We have added information on data availability line 257-267: "The whole genome sequencing data for this study (fastq files of all short read sequencing analyzes and five of the long read assemblies of the nine selected strains) have been deposited in the European Nucleotide Archive (ENA) at EMBL-EBI under accession number PRJEB59168 (https://www.ebi.ac.uk/ena/browser/view/PRJEB59168). The remaining four long read assemblies have been deposited in GenBank, National Institutes of Health (NIH), BioProject number PRJNA982791 (GenBank Overview (nih.gov)), accession numbers SAMN35714885 (ZNZ13L78), SAMN35713968 (ZNZ17F60), SAMN35714917 (ZNZ55M142), and SAMN35714939 (ZNZ57M158). All other relevant data are within the manuscript, with the accession numbers of the genomes and additional information listed in the Supplementary information (Supplementary 1 Table)."
We have corrected the affiliation of authors Annette Onken, Nina Langeland, Kristine Mørch, and Bjørn Blomberg to "National Centre for Tropical Infectious Diseases, Haukeland University Hospital, Bergen, Norway".

1. Onken A, Said AK, Jorstad M, Jenum PA, Blomberg B. Prevalence and Antimicrobial Resistance of Microbes Causing Bloodstream Infections in Unguja, Zanzibar. PloS one. 2015;10(12):e0145632.
2. Ingle DJ, Nair S, Hartman H, Ashton PM, Dyson ZA, Day M, et al. Informal genomic surveillance of regional distribution of Salmonella Typhi genotypes and antimicrobial resistance via returning travellers. PLoS neglected tropical diseases. 2019;13(9):e0007620.

3. Hendriksen RS, Leekitcharoenphon P, Lukjancenko O, Lukwesa-Musyani C, Tambatamba B, Mwaba J, et al. Genomic signature of multidrug-resistant Salmonella enterica serovar typhi isolates related to a massive outbreak in Zambia between 2010 and 2012. Journal of clinical microbiology. 2015;53(1):262-72.
4. Phan MD, Kidgell C, Nair S, Holt KE, Turner AK, Hinds J, et al. Variation in Salmonella enterica serovar typhi IncHI1 plasmids during the global spread of resistant typhoid fever. Antimicrob Agents Chemother. 2009;53(2):716-27.
5. Kariuki S, Revathi G, Kiiru J, Mengo DM, Mwituria J, Muyodi J, et al. Typhoid in Kenya is associated with a dominant multidrug-resistant Salmonella enterica serovar Typhi haplotype that is also widespread in Southeast Asia. Journal of clinical microbiology. 2010;48(6):2171-6.
6. Holt KE, Phan MD, Baker S, Duy PT, Nga TV, Nair S, et al. Emergence of a globally dominant IncHI1 plasmid type associated with multiple drug resistant typhoid. PLoS neglected tropical diseases. 2011;5(7):e1245.
7. Wong VK, Baker S, Pickard DJ, Parkhill J, Page AJ, Feasey NA, et al. Phylogeographical analysis of the dominant multidrug-resistant H58 clade of Salmonella Typhi identifies inter- and intracontinental transmission events. Nature genetics. 2015;47(6):632-9.
8. Ashton PM, Nair S, Dallman T, Rubino S, Rabsch W, Mwaigwisya S, et al. MinION nanopore sequencing identifies the position and structure of a bacterial antibiotic resistance island. Nature biotechnology. 2015;33(3):296-300.
9. Carey ME, Dyson ZA, Ingle DJ, Amir A, Aworh MK, Chattaway MA, et al. Global diversity and antimicrobial resistance of typhoid fever pathogens: Insights from a meta-analysis of 13,000 Salmonella Typhi genomes. eLife. 2023;12.
10. Threlfall EJ, Ward LR, Skinner JA, Smith HR, Lacey S. Ciprofloxacin-resistant Salmonella typhi and treatment failure. Lancet. 1999;353(9164):1590-1.
